# *Dictyostelium discoideum* as a Model for Investigating Neurodegenerative Diseases

**DOI:** 10.3389/fncel.2021.759532

**Published:** 2021-10-27

**Authors:** Holly N. Haver, K. Matthew Scaglione

**Affiliations:** ^1^Department of Molecular Genetics and Microbiology, Duke University, Durham, NC, United States; ^2^Department of Neurology, Duke University, Durham, NC, United States; ^3^Duke Center for Neurodegeneration and Neurotherapeutics, Duke University, Durham, NC, United States

**Keywords:** *Dictyostelium discoideum*, neurodegeneration, model organism, polyglutamine (polyQ) diseases, Alzheimer’s disease, Parkinson’s disease

## Abstract

The social amoeba *Dictyostelium discoideum* is a model organism that is used to investigate many cellular processes including chemotaxis, cell motility, cell differentiation, and human disease pathogenesis. While many single-cellular model systems lack homologs of human disease genes, *Dictyostelium’s* genome encodes for many genes that are implicated in human diseases including neurodegenerative diseases. Due to its short doubling time along with the powerful genetic tools that enable rapid genetic screening, and the ease of creating knockout cell lines, *Dictyostelium* is an attractive model organism for both interrogating the normal function of genes implicated in neurodegeneration and for determining pathogenic mechanisms that cause disease. Here we review the literature involving the use of *Dictyostelium* to interrogate genes implicated in neurodegeneration and highlight key questions that can be addressed using *Dictyostelium* as a model organism.

## Introduction – Model Organisms for Neurodegenerative Diseases

As the human population ages, neurodegenerative diseases are becoming increasingly prevalent. Neurodegenerative diseases are characterized by neuronal dysfunction and degeneration. This dysfunction and degeneration can be caused by both sporadic and genetic causes, affect different subsets of neurons, and have varied pathological hallmarks. Neurodegenerative disease progression inevitably leads to disability and death, and for nearly all these diseases there is a lack of curative treatments. Therefore, understanding the molecular mechanisms that drive neurodegenerative diseases is important for developing therapies.

One way to investigate the mechanisms that drive neurodegenerative diseases is by utilizing model organisms. Neurodegenerative diseases have been studied in a wide variety of model systems ranging from yeast to non-human primates. Model organisms like yeast, worms, flies, and zebrafish can be utilized to investigate the basic pathology of neurodegenerative diseases and are useful models that are inexpensive and easy to genetically manipulate (Miller-Fleming et al., 2008; van Ham et al., 2009; Gama Sosa et al., 2012; Andrews et al., 2016; Suresh et al., 2018). These models are advantageous for determining basic pathophysiological mechanisms, but lack key aspects of the higher eukaryotes that can affect disease pathogenesis such as myelination of neurons and immune function; this means that they do not fully recapitulate human diseases (Gama Sosa et al., 2012). Other models include mammalian cell culture including primary neuronal cultures and induced pluripotent stem cell (iPSC)-derived neuronal cultures. Primary neuronal cultures and iPSCs are advantageous because they are more similar to the human brain than non-mammalian models (Lopes et al., 2017; Slanzi et al., 2020). However, these models do have some drawbacks including the fact they are more fetal-like in nature, and thus may not be an ideal model for age-related neurodegenerative diseases (Kumar et al., 2018; Slanzi et al., 2020). More complex model organisms, like rodents and non-human primates, have more similar neuroanatomy and neurophysiology to the human brain, and phenotypes associated with various neurodegenerative diseases can be recapitulated in these models (Emborg, 2017; Dawson et al., 2018; Suresh et al., 2018). In addition, more complex models show disease progression with aging, which is harder to observe in simpler systems with shorter life spans (Emborg, 2017; Dawson et al., 2018). Rodent and primate models are also essential for testing the feasibility and safety of therapeutics prior to clinical trials (Emborg, 2017; Dawson et al., 2018; Suresh et al., 2018). Therefore, each model organism has strengths and weaknesses that must be taken into consideration when investigating neurodegenerative diseases.

One major unanswered question for many neurodegenerative diseases is: What is the normal function of the proteins that cause disease? This is an important question because one possibility is that neurodegenerative diseases are caused by either a loss of or a toxic gain of protein function. One way to delineate the normal function of proteins that cause neurodegenerative diseases is through utilizing non-mammalian model systems. Many non-mammalian model systems have numerous benefits including simpler genomes, decreased genetic redundancy, ease of genetic manipulation, shorter generation times, and scalability for high throughput genetic and pharmacological studies (van Ham et al., 2009; Gama Sosa et al., 2012; Bozarro, 2013; Suresh et al., 2018). These advantages allow for more rapid investigation into mechanisms that cause neurodegeneration that cannot be accomplished as easily in mammalian model systems.

**FIGURE 1:**
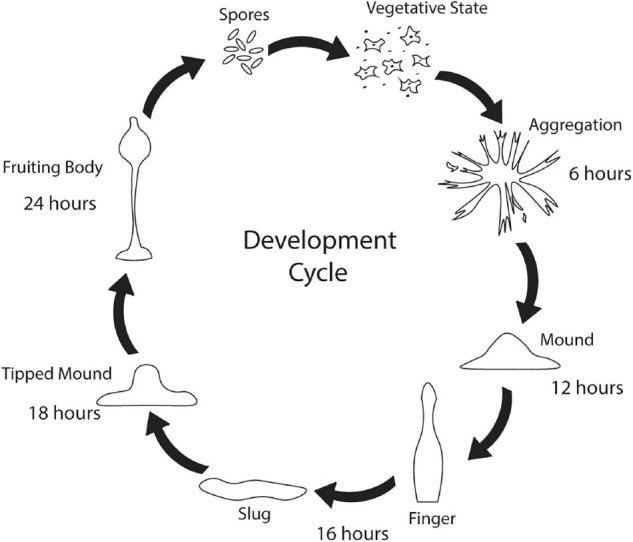
*Dictyostelium’s* developmental cycle. Under stress conditions such as starvation, unicellular *Dictyostelium* emits a cAMP signal causing individual cells to culminate, forming aggregates after 6 h. After 12 h, the aggregates begin to form mounds which turn into finger/slug structures around 16 h. The slug carries the *Dictyostelium* cells to the top of the forest floor where it forms a tipped mound around 18 h. After 24 h, a fruiting body consisting of a spore filled sorus is hoisted by a stalk. Fruiting bodies are then carried off by wildlife and can germinate and propagate elsewhere.

While *Saccharomyces cerevisiae, Caenorhabditis elegans*, and *Drosophila melanogaster* are popular non-mammalian systems for neurodegenerative disease research, *Dictyostelium discoideum* (*Dictyostelium*) is another simple organism that is useful as a model to investigate mechanisms that cause disease. *Dictyostelium* is a soil-dwelling amoeba found throughout the world. *Dictyostelium* consumes bacteria, however, when bacteria are depleted, *Dictyostelium* undergoes a developmental cycle transitioning from a unicellular amoeba to a multicellular fruiting body (Figure 1). This developmental process makes *Dictyostelium* an ideal model organism for investigating numerous biological processes including chemotaxis, cell differentiation, and tissue formation. In addition to these cellular processes, *Dictyostelium* is also a useful model of human diseases, including neurodegenerative diseases. Surprisingly, despite the lack of a nervous system, *Dictyostelium’s* genome encodes a number of genes that cause neurodegenerative diseases (Table 1). This is a substantial increase in genes that cause neurodegeneration compared to *S. cerevisiae*, another single-celled organism commonly utilized for genetic screening and elucidation of gene function (Table 2). This makes *Dictyostelium* a good model organism for identifying the normal function of genes that cause neurodegeneration and for identifying how mutations in these genes may disrupt function.

**TABLE 1:** Human neurodegenerative disease proteins and their *Dictyostelium* homologs.

**Human Protein**	***Dictyostelium* Protein**	**Neurodegenerative disease**	**Human Protein**	***Dictyostelium* Protein**	**Neurodegenerative disease**
PSEN1	Q54DE8	Alzheimer’s disease	HTT	Q76P42	Huntington’s disease
PSEN2	Q54ET2				
ABCA7	Q8T6J5		CLN1	Q54N35	Neuronal ceroid lipofucinosis/batten disease
PLD3	Q54SA1		CLN2	Q55CT0	
CD2AP	Q54F41		CLN3	Q54F25	
BIN1	Q54IA6/Q54NT2		CLN4	Q54GP6/Q86KX9	
INPP5D	Q54W40		CLN5	Q553W9	
CELF1	Q54EJ3		CLN7	Q8T2G9	
PFK	P90521		CLN10	O76856	
PK	Q54RF5				
CLU	O15818		SACS	Q55EK5/Q55EK4/Q55EK6	ARSACS
UCHL1 DJ-1	Q54T48 Q54MG7	Parkinson’s disease	PEX1 PEX3	Q54GX5 Q54U86	Infantile refsum disease
ATP13A2	Q54NW5/Q54 × 63		PEX6 PEX12	Q54CS8 Q54N40	
GIGYF2	Q54WZ1		PHYH	Q54BP2	Refsum disease
VPS35	Q54C24				
EIF4G1	Q553R3		PEX7	Q54WA3	
PGK1	Q9GPM4				
VPS13C GCH1	Q55FG3 Q94465		VPS13a	Q54LB8/Q555C6	Chorea-acanthocytosis
SOD1	Q54G70	Amyotrophic lateral sclerosis	SMPCD1	Q54C16	Niemann-Pick disease
UBQLN2	Q9NIF3		NPC1	Q551C5/Q9TVK6	
KIF5A	Q54UC9				
CHCHD10	Q54BU1		TSEN2	Q54RX5	Pontocerebellar hypoplasia
TUBA4A	P32255		TSEN34	Q556W4	
ANXA11	P24639		TSEN54	Q54ND7	
PGK1	Q9GPM4		RARS2	Q558Z0	
ATXN2	Q55DE7	Spinocerebellar ataxia	PDHA1	Q54C70	Pyruvate dehydrogenase deficiency
SPTBN2	Q54DR3		DLAT	P36413	
ATXN10	Q55EI6		PDHB	Q86HX0	
PPP2R2B	Q54Q99				
ITPR1	Q9NA13		HEXB	P13723/Q54SC9	Sandoff disease
TBP	P26355				
EEF2	P151122/Q54JV1		HEXA	P13723/Q54SC9	Tay-Sachs disease
AFGL2	Q75JS8				
ELOVL4	Q55BY4		ERCC6	Q54TY2	Cockayne syndrome
NOP56	Q54MT2				
AT2B1	P54678/Q54HG6		ABCA1	Q8T5Z7	Tangier’s disease
VPS13D	Q55FG3				
SCYL1	Q55GS2		FXN	Q54C45	Friedreich’s ataxia

**TABLE 2:** Comparison of neurodegenerative disease genes present in *Dictyostelium* versus *S. cerevisiae*.

**Neurodegenerative disease**	**Gene Present in *Dictyostelium***	**Present in *S. cerevisiae***	**Neurodegenerative Disease**	**Gene Present in *Dictyostelium***	**Present in *S. cerevisiae***
Alzheimer’s disease	ABCA7	+	ARSACS	SACS	−
	PLD3	−			
	PSEN1	−	Neuronal ceroid lipofucinoses (Batten diseases)	CLN1	−
	PSEN2	−		TPP (CLN2)	−
	CD2AP	−		CLN3	+
	BIN1	−		CLN4	−
	INPP5D	−		CLN5	−
	CELF1	+		CLN7	−
				CLN10	+
Parkinson’s disease	UCHL1	+	Infantile osteopetrosis	CLCN7	−
	DJ-1	−			
	ATP13A2	+			
	GIGYF2	+	Infantile refsum disease	PEX1	−
	VPS35	+		PEX3	+
	EIF4G1	−		PEX6	+
				PEX12	+
Amyotrophic lateral sclerosis	SOD1	+			
	KIF5A	−		PHYH	−
	CHCHD10	+			
	TUBA4A	+	Chorea acanthocytosis	VPS13a	+
	ANXA11	−	Niemann Pick diseases	SMPD1	−
Huntington’s disease	HTT	−		NPC1	+
Spinocerebellar ataxia	ATXN2	−	Pontocerebellar hypoplasia	TSEN2	−
	ATXN10	−		TSEN34	+
	TBP	+		TSEN54	−
	PPP2R2B	+		RARS2	+
	ITPR1	−			
	AFG3L2	+	Pyruvate dehydrogenase deficiency	PDHA1	+
	ELOVL4	+		PDHB	+
	NOP56	+		DLAT	+
Spinocerebellar ataxia, X-linked	AT2B1	+	Sandhoff disease	HEXB	−
Spinocerebellar ataxia autosomal recessive	VPS13D	−	Tay-Sachs disease	HEXA	−
	SCYL1	+			
	SPTBN2	−	Cockayne syndrome	ERCC6	+
	EEF2	+			

**FIGURE 2:**
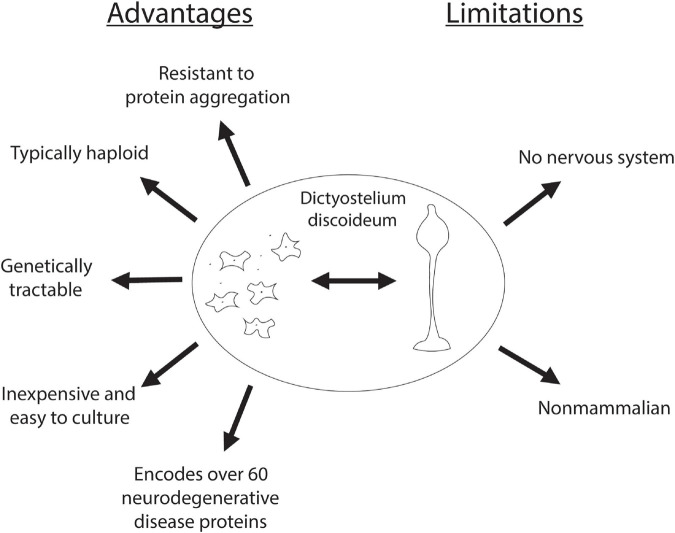
Advantages and limitations of *Dictyostelium* as a model organism for neurodegenerative diseases. Advantages of using *Dictyostelium* as a model for neurodegenerative diseases are listed on the *left*, and its limitations are listed on the *right*.

In addition to encoding for genes that cause neurodegeneration, *Dictyostelium* also has numerous technical advantages that make it an advantageous model system. One of these advantages is that *Dictyostelium* is typically haploid, simplifying gene disruption, however, it does have both sexual and parasexual cycles, enabling the investigation of gene complementation (Loomis, 1987; Bloomfield et al., 2010; Bozarro, 2013). In addition, *Dictyostelium* is inexpensive and easy to culture, making it an accessible model organism. *Dictyostelium* is also advantageous in that as an amoeba it is a single cell type, reducing the complexity associated with multiple cell types commonly found with cultured mammalian neurons. Finally, there are several genetic screening protocols established making *Dictyostelium* an attractive model organism for the discovery of gene function. In addition to these advantageous properties there are limitations associated with investigating neurodegenerative diseases. Most notably, *Dictyostelium* lacks a nervous system and thus does not have the complexity found in the human brain. Additionally, neurons are long-lived post-mitotic cells whereas *Dictyostelium* are rapidly dividing (Figure 2). However, despite these drawbacks, *Dictyostelium* has proven to be a useful model organism for probing the function and dysfunction of proteins implicated in neurodegenerative diseases.

## Investigating Neurodegeneration With *Dictyostelium*

*Dictyostelium* is a powerful model organism for understanding the underlying function of genes that are implicated in neurodegeneration. *Dictyostelium* continues to be utilized to interrogate the normal function of genes and to determine how disease-causing mutations alter cellular processes. This information can then be used to validate that these genes disrupt similar pathways in neurons and in the mammalian brain. Here we highlight significant findings in the field of neurodegeneration made in *Dictyostelium* and discuss opportunities for further utilization of *Dictyostelium* as a model organism to interrogate neurodegenerative diseases.

### Alzheimer’s Disease

**FIGURE 3:**
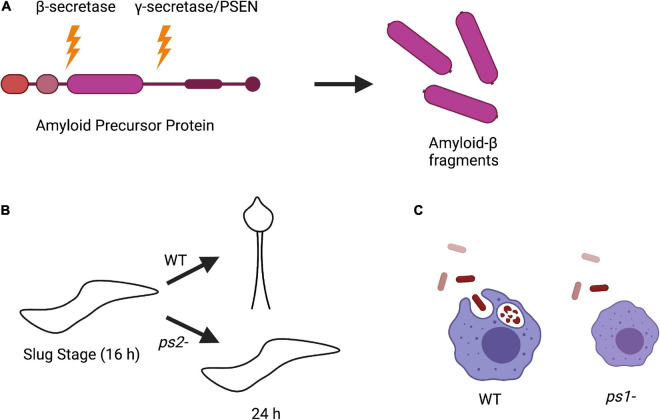
Amyloid precursor protein (APP) processing and phenotypes of *Dictyostelium PSEN* knockout strains. **(A)** Processing of APP in human cells begins with initial cleavage by a β-secretase enzyme, followed by cleavage by presenilin in the γ-secretase complex to produce amyloid-β fragments. **(B)** Knockout of PSEN2 (*ps2*-) in *Dictyostelium* causes impaired ability to differentiate into pre-stalk cells during development and are unable to sporulate compared to wild-type (WT) cells. **(C)** Knockout of PSEN1 (*ps1*-) in *Dictyostelium* results in smaller cell size and defective phagocytosis compared to WT.

Alzheimer’s disease (AD) is the most common neurodegenerative disease. The pathological hallmarks of AD are the accumulation of extracellular amyloid-β (Aβ) plaques and intracellular tau neurofibrillary tangles. Under normal conditions, the amyloid precursor protein (APP) is processed by β-secretases, such as β-secretase 1 (BACE1), and γ-secretases to yield APP C-terminal fragments of different lengths. BACE1 initially cleaves the N-terminal region of APP, and the γ-secretase complex cleaves the C-terminal region. Presenilin (PSEN) 1 or 2 serves as the catalytic subunit of γ-secretase and is involved in processing APP into Aβ peptides (Figure 3A; De Strooper et al., 1998, 1999; Struhl and Greenwald, 1999; Wolfe et al., 1999; Esler et al., 2000; Li et al., 2000). Under normal conditions, the most abundant Aβ peptide is Aβ40, with very little of the toxic Aβ42 fragment present. However, in AD there is an alteration of APP cleavage leading to an increased Aβ42/Aβ40 ratio. This leads to the formation of Aβ aggregates that are thought to be an initial step in AD pathogenesis. Because the Aβ42/Aβ40 ratio is increased, and over 150 missense mutations in *PSEN1/2* have been linked to AD pathogenesis, a large amount of research has investigated the function of the γ-secretase complex in the context of AD (De Strooper et al., 2012; Haass et al., 2012; Mucke, 2012). Therefore, model systems that interrogate the γ-secretase complex and PSENs are useful for understanding the initial steps of AD pathogenesis.

Interestingly, while *Dictyostelium* does not encode for a β-secretase or APP, it does express a divergent form of the γ-secretase complex. Homologous genes have been identified in *Dictyostelium* for all components of the γ-secretase complex including *PSEN1* and *PSEN2*. To investigate the biological function of *PSEN1* and *PSEN2*, knockout strains (*ps1*^–^ and *ps2*^–^, respectively) were developed. *ps2*^–^ cells had developmental defects, could not differentiate into pre-stalk cells during the slug stage, and were unable to sporulate, suggesting that PSEN2 γ-secretase complex activity is required for cell differentiation (Figure 3B; McMains et al., 2010). This is consistent with the observation that PSEN γ-secretase activity is involved in neuronal differentiation (Baumeister, 1999; McMains et al., 2010; van Tijn et al., 2011). On the other hand, deletion of *PSEN1* resulted in a reduced phagocytic ability, consistent with data in mammalian cells that PSENs are present in lysosomal membranes (Figure 3C; McMains et al., 2010; Myre, 2012). The phenotypic differences between *ps1*- and *ps2*- *Dictyostelium* cells also strengthen the hypothesis that there are distinct PSEN γ-secretase complexes that serve different biological functions (Gu et al., 2004; Myre, 2012).

In addition to PSEN1 and 2 regulating phagocytosis and development, respectively, work has also been done to identify functions of additional components of the γ-secretase complex including Aph-1 and nicastrin (Ncstn) (Sharma et al., 2019). It was identified that *Dictyostelium* cells lacking Aph-1, Ncstn, or both PSEN proteins caused a significant decrease in micropinocytosis, indicating a role for these γ-secretase components in endocytosis. Deletion of Aph-1 or both PSEN proteins also caused a decrease in phagosomal proteolysis, an increase in the rate of autophagosome acidification, a decrease in the number of but increase in the size of Atg8-positive structures, and a severe decrease in autophagic flux. These results suggest that Aph-1 and PSEN1/2 regulate autophagy. Finally, it was observed that the large Atg-8-positive structures in Aph-1 and PSEN knockout cells contained high-molecular-weight ubiquitinated protein, consistent with an impairment in autophagic degradation. These phenotypes were rescued by the overexpression of either *Dictyostelium* or human catalytically inactive PSEN, consistent with PSENs and the γ-secretase complex playing non-proteolytic roles in both endocytosis and autophagy pathways (Sharma et al., 2019).

In addition to identifying the role of the γ-secretase complex in *Dictyostelium* biology, it was also found that wild-type *Dictyostelium* cells expressing human APP process APP in a similar manner as human cells, producing both Aβ40 and Aβ42 fragments. This process is dependent on the γ-secretase complex, as cells lacking components of the γ-secretase complex are unable to process APP (McMains et al., 2010). This is an intriguing observation because *Dictyostelium* does not encode for a β-secretase to initiate cleavage, raising the question of how *Dictyostelium* initially processes APP. The absence of a β-secretase should prevent the initial cleavage of APP and halt further processing. This means that *Dictyostelium* has a way of processing APP either without N-terminal cleavage or by another unknown enzyme that may initiate processing. Identifying how *Dictyostelium* initiates APP cleavage may provide novel insight into currently unknown roles of the γ-secretase complex in AD. Additionally, due to the ability to perform genetic screens, *Dictyostelium* may be a powerful model organism for identifying novel modulators of APP processing.

### Parkinson’s Disease

Parkinson’s disease (PD) is the second-most prevalent neurodegenerative disease caused by the loss of dopaminergic neurons in the substantia nigra. This occurs, at least in part, due to the formation and accumulation of protein aggregates called Lewy bodies within the neuron’s cytoplasm. In PD, neurons have numerous problems including oxidative stress, mitochondrial dysfunction, synaptic dysfunction, and inhibition of protein quality control pathways (Cook et al., 2012; Picconi et al., 2012; Dias et al., 2013; Park et al., 2018). There are many proteins that have been implicated in PD pathogenesis, but only α-synuclein, DJ-1, and LRRK2 have been investigated in *Dictyostelium* thus far.

#### α-Synuclein

The α-synuclein protein is highly expressed in the brain, especially in dopaminergic neurons, and several α-synuclein mutations have been mapped to familial cases of PD (Stefanis, 2012). Although *Dictyostelium* does not encode a homolog of the α-synuclein gene, one study was performed to determine the effect of exogenous expression of α-synuclein in *Dictyostelium*. Expression of either wild-type or mutant α-synuclein resulted in impaired phototaxis and thermotaxis, altered fruiting body morphology, and decreased the rate of phagocytosis, consistent with a potential role for α-synuclein in disrupting mitochondrial function in *Dictyostelium* (Fernando et al., 2020). However, the addition of mutant or wild-type α-synuclein increases some or all aspects of mitochondrial respiration, respectively, which is contrary to the other mitochondrial phenotypes previously observed (Fernando et al., 2020). In the future, additional studies of the effects of α-synuclein expression on mitochondrial function in *Dictyostelium* may help clarify α-synuclein’s role in mitochondrial biology.

In addition to potentially disrupting mitochondrial function, α-synuclein also affects normal cellular functions of *Dictyostelium*, suggesting that it may exert some toxicity on *Dictyostelium*. The cause of this toxicity is unknown and further investigation may shed light on how α-synuclein exerts its toxicity. The phenotypes observed by expressing both wild-type and mutant α-synuclein did encompass some of the phenotypes required for mitochondrial dysfunction, suggesting that *Dictyostelium* may have mechanisms to suppress α-synuclein-induced toxicity. If so, *Dictyostelium* could be utilized to identify novel suppressors of mitochondrial dysfunction that could play a protective role in PD.

#### DJ-1

Another protein implicated in PD pathogenesis is DJ-1, a small, dimeric protein that is most highly expressed in cells with high energy demands, such as neurons. Cells with high energy demand typically have high levels of reactive oxygen species, and one function of DJ-1 is to protect cells from oxidative stress (Wagenfeld et al., 1998; Kinumi et al., 2004; Taira et al., 2004; Kim et al., 2005; Meulener et al., 2005; Wilson, 2011; Jain et al., 2012; Ottolini et al., 2013; Batelli et al., 2015; Chunna and Pu, 2017; Eberhard and Lammert, 2017; Kawate et al., 2017; Kiss et al., 2017; Smith and Wilson, 2017; Catazaro et al., 2018). DJ-1 has also been implicated to act as a chaperone, modulating the toxicity and misfolding of both α-synuclein and mutant huntingtin (Batelli et al., 2008; Wang et al., 2011; Sajjad et al., 2014; Zondler et al., 2014). There have been many DJ-1 mutations identified in PD, some of which have been clearly linked to PD pathogenesis by disrupting DJ-1 dimerization (Wilson et al., 2003; Gorner et al., 2007; Malgieri and Eliezer, 2008; Ramsey and Giasson, 2010). While DJ-1 is highly expressed in astrocytes of the frontal cortex and substantia nigra in both control and PD brains, analysis of PD brains showed decreased levels of both mRNA and protein across the entire brain (Bandopadhyay et al., 2004; Kumaran et al., 2009). Currently, the role of DJ-1 in both healthy and PD cells, as well as how it contributes to PD pathogenesis, is unknown, although it has been proposed to be linked to mitochondrial dysfunction (Repici and Giorgini, 2019).

*Dictyostelium’s* genome encodes a homolog of *DJ-1* that has been utilized to investigate its normal biological role (Chen et al., 2017). In *Dictyostelium*, DJ-1 is located in the cytoplasm, and deletion of *DJ-1* results in growth defects, but not mitochondrial dysfunction (Chen et al., 2017). While deletion of DJ-1 did not result in a mitochondrial phenotype, transient knockdown of DJ-1 slightly increased mitochondrial respiration whereas overexpression of DJ-1 inhibited respiration (Chen et al., 2017). The conflicting results between the *DJ-1* knockout and transient knockdown strains could be due to genetic compensation that may occur in the knockout but not in the knockdown (El-Brolosy and Stainier, 2017). The conflicting findings between deletion of *DJ-1* versus knockdown of *DJ-1* suggest further work is warranted to understand the potential role of DJ-1 in regulating mitochondrial function in *Dictyostelium*.

A follow-up study decided to investigate the function of DJ-1 under oxidative stress conditions, as it could differ compared to normal cellular conditions (Chen et al., 2021). Oxidative stress on wild-type *Dictyostelium* cells resulted in inhibition of mitochondrial respiration and impairment of phagocytosis in an AMPK-dependent manner, which worsens during *DJ-1* knockdown. Oxidative stress in combination with *DJ-1* loss also leads to worsened defects in phototaxis, morphogenesis, and growth, also in an AMPK-dependent manner. These phenotypes all coincide with the *Dictyostelium* model of mitochondrial dysfunction. These data suggest therefore that the presence of DJ-1 in its oxidized form is protective of effects caused by oxidative stress and AMPK hyperactivity (Chen et al., 2021). Further studies should be performed to better understand the role of DJ-1 in both normal and oxidative conditions. Once the role of DJ-1 is well defined in *Dictyostelium*, this model system would be a powerful tool to interrogate dysfunction caused by known PD pathways and could increase our understanding of how mutations in DJ-1 result in PD pathology.

#### Leucine-Rich Repeat Kinase 2

Leucine-rich repeat kinase 2 (*LRRK2*) is a commonly mutated gene in both sporadic and inherited forms of PD. *LRRK2* encodes a large protein with GTPase, kinase, and scaffolding domains (Bosgraaf and van Haastert, 2003). It is a member of the Roco protein family, having Roc (Ras of complex) and COR (C-terminus of Roc) domains, along with a leucine-rich repeat (LRR) at its N-terminus (Rui et al., 2018). LRRK2 is a cytoplasmic protein that associates with intracellular membranes, such as the endoplasmic reticulum, and vesicular structures (Hatano et al., 2007; Alegre-Abarrategui et al., 2009). LRRK2 is expressed in many tissues, but it is highly expressed in dopaminergic neurons of the mammalian brain (Biskup et al., 2006; Galter et al., 2006; Higashi et al., 2007). In PD, LRRK2 kinase activity is often increased, which in turn has many downstream effects including impaired dopamine neurotransmission, dopaminergic neuronal cell death, protein synthesis and degradation defects, increased inflammatory response, and oxidative damage (Liou et al., 2008; Carballo-Carbajal et al., 2010; Chen et al., 2012; Maekawa et al., 2016; Rui et al., 2018). Therefore, understanding the cellular pathways that regulate LRRK2’s kinase activity is warranted.

Roco proteins were originally discovered in *Dictyostelium*, and eleven Roco proteins have been identified in *Dictyostelium*, whereas only four have been identified in vertebrates, including humans (Bosgraaf et al., 2002; Bosgraaf and van Haastert, 2003). The Roco4 protein in *Dictyostelium* has the same domain architecture as LRRK2 (Kortholt et al., 2012). In *Dictyostelium*, Roco4’s Roc domain is essential for kinase activity, while the COR domain functions for protein dimerization. Point mutations within the Roco4 Roc or kinase domains inactivate it but do not lead to loss of GTP-binding (Kortholt et al., 2012). Furthermore, PD-related mutations in Roco4 revealed correlating decreases in GTPase activity and increasing kinase activity except for L1180T (LRRK2 I2020T), which shows reduced kinase activity like its LRRK2 counterpart (Jaleel et al., 2007; Ohta et al., 2010; Kortholt et al., 2012). Functionally, deletion of Roco4 or LRRK2 or expression of mutant forms of LRRK2 in *Dictyostelium* and in human macrophages indirectly leads to mitochondrial dysfunction (Rosenbusch et al., 2021). Therefore, Roco4 is an attractive candidate for investigating the role of LRRK2 in normal physiology and in PD. While Roco4 is similar architecturally to LRRK2, it has not been confirmed as a true homolog of LRRK2. Further work must be done to determine if Roco4 and LRRK2 are homologs. One way to do this would be attempting to rescue a Roco4 *Dictyostelium* knockout with LRRK2. If the knockout phenotypes are alleviated, this would suggest similar cellular roles for both Roco4 and LRRK2.

### Huntington’s Disease

Huntington’s disease (HD) is caused by the expansion of a CAG trinucleotide repeat in the coding region of the huntingtin (*Htt*) gene. This CAG expansion is then translated into a polyglutamine (polyQ) tract in the huntingtin protein. Long polyQ tracts (>35Q) result in the misfolding and aggregation of the huntingtin protein. This aggregation process is thought to be one of the mechanisms that lead to disease pathogenesis. In addition to aggregation, both loss of normal huntingtin function and toxic gain of function have been proposed to contribute to toxicity (Ross, 1995; Perutz, 1999; Imarisio et al., 2008). Therefore, understanding the function of the huntingtin protein is important for understanding how polyQ tract expansion causes defects in huntingtin function resulting in HD pathogenesis. One way the normal function of the huntingtin protein has been investigated is by using model organisms. *Htt* has homologs in many model systems including mice, zebrafish, *Drosophila*, and *Dictyostelium*. Studies in these organisms suggest that huntingtin plays an important role in development (Duyao et al., 1995; Nasir et al., 1995; Zeitlin et al., 1995; Lumsden et al., 2007; Zhang et al., 2009). Therefore, *Dictyostelium* provides an excellent model organism to interrogate huntingtin function.

In *Dictyostelium* the huntingtin protein has similar features to that of human huntingtin (Chisholm et al., 2006; Myre, 2012). Interestingly, unlike human huntingtin, *Dictyostelium* huntingtin does not have a polyQ tract encoded in exon 1. However, it does have a short (∼19Q) polyQ tract further along in its amino acid sequence that is composed of mostly CAA trinucleotide repeats rather than CAG (Insall, 2005; Myre et al., 2011). Because the polyQ tract is not in a similar position as in humans, and because *Dictyostelium* has a repeat-rich genome/proteome, it is unclear if this polyQ tract plays a similar role to the polyQ tract found in the human huntingtin protein. To elucidate the function of *Dictyostelium’s* huntingtin protein, *Htt-* cells were generated. *Htt*- cells are viable but have many subtle phenotypes, suggesting that huntingtin is involved in multiple cellular processes (Myre et al., 2011). *Htt*- cells placed in a low ionic strength phosphate buffer became round and lacked membrane extensions due to a reduction in F-actin. This is consistent with mammalian studies showing that huntingtin regulates neurological processes like actin-rich dendritic spine formation and membrane branching and explains defective actin remodeling in HD patient cells (Ferrante et al., 1991; Dent et al., 2011; Munsie et al., 2011; Myre et al., 2011). *Htt*- cells cultured in the absence of exogenous Ca^2+^ cannot initiate cAMP-induced Ca^2+^ transients, thus impairing cAMP relaying and chemotaxis. This suggests a role for huntingtin in *Dictyostelium* chemotaxis and development (Myre et al., 2011). *Htt*- cells also failed to populate the pre-spore region of the slug and therefore did not develop into spores, indicating a need for huntingtin to make viable spores, consistent with huntingtin regulating cell fate during development (Myre et al., 2011). This data supports other vertebrate studies where similar cell fate defects have been observed in the absence of huntingtin (Reiner et al., 2001; Henshall et al., 2009). The research performed in *Dictyostelium* on huntingtin further supports vertebrate research that huntingtin is a multifunctional protein involved in many cellular processes. Because the huntingtin protein in *Dictyostelium* does not contain a polyglutamine tract it could be an interesting model system to probe the functional role of polyQ. It would be interesting to determine the effects that a normal or expanded polyQ tract in exon 1 of *Dictyostelium* huntingtin would have in wild-type or *Htt*- *Dictyostelium* cells. It would also be interesting to determine if human huntingtin with either a normal or expanded polyQ tract would rescue the phenotypes of *Htt*- *Dictyostelium* cells. Future studies such as these could allow us to better understand similarities between huntingtin homologs as well as help delineate differences in huntingtin’s function in its expanded form.

### Neuronal Ceroid Lipofuscinosis (Batten Disease)

Neuronal ceroid lipofuscinosis (NCL), or Batten Disease, encompasses a growing class of debilitating neurodegenerative lysosomal storage diseases. NCLs are also the most common neurodegenerative diseases seen in children (Mole and Cotman, 2015). These diseases are caused by the accumulation of ceroid lipopigments in the lysosomes, along with many NCL-associated proteins (Jalanko and Braulke, 2009). There are thirteen ceroid lipofuscinosis neuronal (CLN) genes/proteins implicated in this class of diseases, with mutations in different genes giving way to different NCLs. Despite their role in NCLs, the normal functions of CLN proteins remain unclear.

#### CLN3

CLN3 is one of 13 proteins that when mutated cause NCL. Mutations in the *CLN3* gene cause the most common subclass of NCLs, juvenile NCL (JNCL) (International Batten Disease Consortium, 1995). The *CLN3* gene encodes for the CLN3 protein, a transmembrane protein that localizes to lysosomes, endosomes, and potentially other subcellular membranes (Cotman and Staropoli, 2012; Uusi-Rauva et al., 2012; Kollmann et al., 2013). While CLN3’s precise function is unknown it has been implicated in several cellular processes including lysosomal pH homeostasis, endocytic trafficking, and autophagy (Pearce et al., 1999; Holopainen et al., 2001; Gachet et al., 2005; Cao et al., 2006; Getty and Pearce, 2011).

**FIGURE 4:**
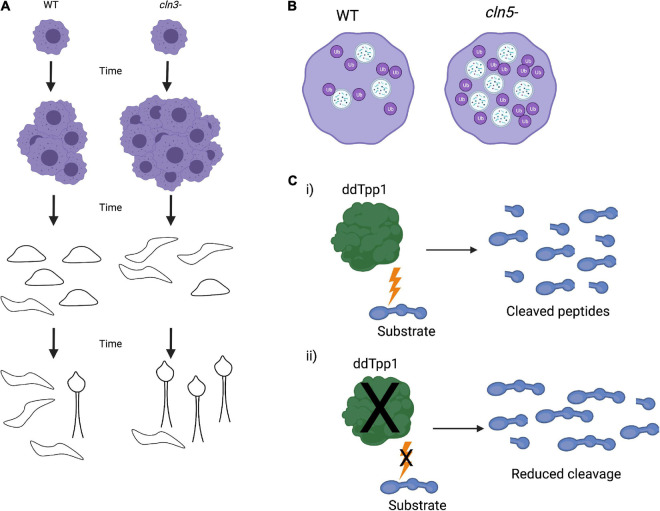
*Dictyostelium* phenotypes of *Cln* knockout strains. **(A)** Knockout of *Cln3* (*cln3*^–^) in *Dictyostelium* leads to an increase in vegetative growth as well as faster slug formation, migration, and fruiting body formation. **(B)** *Cln5* knockout (*cln5*^–^) results in an increase in autophagosome formation and ubiquitination of proteins, indicating upregulated autophagic activity. **(C)** *Dictyostelium* ddTpp1 normally cleaves a synthetic human substrate of human TPP1 **(i)**, but in *tpp1*- cells, cleavage of the substrate is greatly reduced **(ii)**.

*Dictyostelium’s* genome has a homolog for *CLN3* (*Cln3*), which was used as a model to understand its normal function (Huber et al., 2014). In *Dictyostelium*, deletion of *Cln3* results in increased proliferation during vegetative growth. This increase in growth is caused by altered levels of secretory proteins that regulate proliferation signaling. RNAseq revealed that *Cln3* mRNA levels dramatically increase during mid-development. Consistent with a role for *Cln3* at mid-development, *cln3*^–^ cells showed faster slug formation, increased slug migration, and accelerated fruiting body formation, suggesting that Cln3 plays a role in regulating the speed of development (Figure 4A; Rot et al., 2009; Huber et al., 2014). Ca^2+^ chelation was found to restore developmental timing to the rate of WT cells and suppresses abnormal slug migration, indicating that Cln3 also regulates Ca^2+^-dependent developmental events (Huber et al., 2014).

While *cln3*^–^ cells have accelerated fruiting body formation, *cln3*^–^ cells also have a delay in streaming and aggregation due to reduced cell-cell adhesion (Huber et al., 2017). Further investigation into this phenotype determined that Cln3 localizes to the contractile vacuole network and colocalizes with the Golgi marker wheat germ agglutinin. This suggests that Cln3 is involved in both conventional and unconventional secretory pathways during development. Mass spectrometry of wild-type versus *cln3*^–^ *Dictyostelium* cells revealed that the most affected proteins in *cln3*^–^ cells were involved in endocytosis, vesicle-mediated transport, proteolysis, and metabolism, supporting the hypothesis that Cln3 plays a role in secretory pathways (Huber, 2017). Cln3 has also been implicated in *Dictyostelium’s* osmoregulation, and osmoregulatory defects have been observed in mammalian cell models of Batten disease (Stein et al., 2010; Getty et al., 2013; Tecedor et al., 2013; Mathavarajah et al., 2018). Under hypotonic stress, *cln3*^–^ cells show defects in cytokinesis, have reduced viability and impaired spore integrity. Under hypertonic stress, *cln3*^–^ cells also have reduced viability and development is inhibited (Mathavarajah et al., 2018). This indicates Cln3 plays an important role in osmoregulation.

Finally, RNAseq analysis of *cln3*^–^ *Dictyostelium* cells unveiled over 1,000 genes that are differentially expressed during *Dictyostelium* starvation, including many homologs of NCL genes. Loss of Cln3 alters the expression and activity of lysosomal enzymes, increases lysosomal pH, and alters nitric oxide homeostasis (Huber and Mathavarajah, 2019). Upregulation of the *Dictyostelium* homolog of tripeptidyl peptidase 1 (Tpp1) was also observed, in addition to a correlating increase in Tpp1 enzyme activity (Mathavarajah et al., 2018). Autofluorescent storage bodies were observed in starving *Dictyostelium* cells, which were also found in starving *tpp1*- cells, linking Cln3 function to Tpp1 activity (Phillips and Gomer, 2015; Huber and Mathavarajah, 2019). Because Cln3 has conserved function in *Dictyostelium* and human cells, further studies are warranted to fully understand Cln3 function and to determine how mutations in *Cln3* result in disease.

#### CLN5

Another protein implicated in NCLs is CLN5. Mutations in *CLN5* have been linked to late infantile, juvenile, and adult NCLs (Pineda-Trujillo et al., 2005; Cannelli et al., 2007; Xin et al., 2010; Mancini et al., 2015; Simonati et al., 2017). CLN5 localizes to the lysosomal matrix and extracellular space and alters numerous processes including neurogenesis, synaptic recycling, and autophagy (Isosomppi et al., 2002; von Schantz et al., 2008; Schmiedt et al., 2012; Larkin et al., 2013; Moharir et al., 2013; Fabritius et al., 2014; Hughes et al., 2014; Cárcel-Trullols et al., 2015; De Silva et al., 2015; Best et al., 2017; Jules et al., 2017; Leinonen et al., 2017; Uusi-Rauva et al., 2017). However, the precise function of CLN5 and its role in NCLs is unknown.

Similar to Cln3, *Dictyostelium* also expresses a homolog of CLN5 (Cln5). Cln5 localizes to the ER, and both *Dictyostelium* Cln5 and exogenous human CLN5 have glycoside hydrolase activity in *Dictyostelium* cells. Immunoprecipitation and mass spectrometry identified numerous Cln5 interactors, many of which are implicated in NCLs. Some of these interactors include cathepsin D, tripeptidyl peptidase 1, and CDC48 (Huber and Mathavarajah, 2018). Further investigation of Cln5 was found that *cln5*^–^- cells have reduced cell proliferation, cytokinesis, viability, folic acid-mediated endocytosis, and growth in nutrient-limited media. *cln5*^–^ cells develop more rapidly than wild-type cells (McLaren et al., 2021). *cln5*^–^ cells also exhibit impaired spore morphology, germination, and viability. At a cellular level deletion of *cln5* results in an increased number of autophagosomes and ubiquitinated proteins, consistent with increased autophagic activity (Figure 4B). Development in the presence of an autophagy inhibitor impaired the formation of developmental structures, including reduction in slug size (McLaren et al., 2021). These data suggest that Cln5 plays a similar role in both *Dictyostelium* and human biology and may play a role in autophagy. Further work using Cln5 in *Dictyostelium*, including introducing pathogenic NCL mutations, may contribute to better understanding the native function of CLN5 and NCL pathogenesis. These studies could also be expanded into mammalian systems to confirm the findings in *Dictyostelium* and facilitate therapeutic avenues for NCLs.

#### Tripeptidyl Peptidase 1

Another subclass of NCL, late infantile NCL (LINCL), is caused primarily by mutations in tripeptidyl peptidase 1 (TPP1, also known as CLN2), a lysosomal peptidase (Sleat et al., 1997). TPP1 cleaves tripeptides of the N-terminus of proteins with optimal peptidase activity occurring at pH 3.5 (Vines and Warburton, 1998; Sohar et al., 1999). However, its *in vivo* substrates and physiological function are unknown (Phillips and Gomer, 2015). Studies in LINCL fibroblasts revealed that the lysosomal TPP1’s activity is dramatically reduced compared to fibroblasts from unaffected individuals (Vines and Warburton, 1999). While TPP1 is conserved among vertebrates, there are no homologs of TPP1 found among most invertebrate model organisms such as *Drosophila*, *C. elegans, and S. cerevisiae*, limiting the utilization of these model organisms in investigating TPP1 function (Wlodawer et al., 2003; Phillips and Gomer, 2015).

Unlike other lower eukaryotes, *Dictyostelium* does possess a homolog of TPP1, ddTpp1. Deletion of ddTpp1 results in a reduced, but not a complete loss of, the ability to cleave a synthetic substrate of human TPP1 (Figure 4C). Both ddTpp1 and human TPP1 localize to the lysosome when expressed in *Dictyostelium*. *tpp1*- cells display normal vegetative growth but undergo development more rapidly than wild-type cells. Once developed, the fruiting bodies also have a reduced number of spores. Starved *tpp1*- cells form intracellular auto-fluorescent bodies analogous to those found in patients lacking TPP1, and *tpp1*- cells starved of amino acids are smaller in size and have reduced viability, indicating defects in autophagy. Finally, in the presence of chloroquine (a lysosome-perturbing compound), *tpp1*- cells have a highly impaired developmental cycle (Phillips and Gomer, 2015). These data point to ddTpp1 playing roles in both autophagy and *Dictyostelium’s* developmental cycle. Consistent with this, inhibition of autophagy by treating cells with the target of rapamycin (TOR) complex inhibitor rapamycin or by knocking down the upstream activator of the TOR complex Ras homology enriched in brain (RHEB) results in the same phenotypes observed in *tpp1*- cells (Smith et al., 2019). Furthermore, overexpression of RHEB rescues these defects, suggesting that TOR signaling could be responsible for *tpp1*- phenotypes (Smith et al., 2019). It is important to note that knocking out ddTpp1 does not cause a complete loss of substrate cleavage (Phillips and Gomer, 2015). This suggests that there may be other proteases or peptidases present in *Dictyostelium* that could be substituted for ddTPP1. If so, this could mean there may be compensatory cleavage mechanisms in human cells as well. In the future it would be interesting to identify other proteases that can cleave TPP1 substrates in *Dictyostelium*. Identification of other proteins that can cleave TPP1 substrates may lead to novel ideas to guide the design of LINCL therapies in the future.

### Hirano Bodies

Hirano bodies are cytoplasmic protein aggregates that have crystalloid fine rod structures (Cartier et al., 1985; Hirano, 1994). They contain numerous different proteins such as actin, actin-binding proteins, microtubule-associated proteins, tau, C-terminal fragments of APP, and neurofilaments (Hirano, 1994). These inclusions are seen preferentially in the neuronal processes of patients of many neurodegenerative diseases including AD, parkinsonism-dementia, amyotrophic lateral sclerosis (ALS), Creutzfeldt-Jacob disease, and Pick’s disease (Cartier et al., 1985; Hirano, 1994). However, Hirano bodies have also been found in other cell types such as glia, peripheral nerve axons, and extraocular muscles of the eyes (Tomanaga, 1983). While Hirano bodies are associated with neurodegenerative diseases, Hirano bodies also form as a function of age and can be found in the brain of aged people without neurodegenerative diseases (Gibson and Tomlinson, 1997).

*Dictyostelium* does not naturally form Hirano bodies, however, it has been used to model them. A model of Hirano bodies was created in *Dictyostelium* using the *Dictyostelium* actin crosslinking protein called the 34-kDa protein (Maselli et al., 2002). By expressing the C-terminal fragment of the 34-kDa protein (CT) in *Dictyostelium*, the formation of para-crystalline inclusions resembling Hirano bodies was observed. These structures contain ordered assemblies of CT, F-actin, myosin II, cofilin, and α-actinin, typical of human Hirano bodies. Developmental studies performed on *Dictyostelium* expressing CT found that development was delayed by 6 h. In addition to *Dictyostelium*, expressing CT in multiple mammalian cell systems induced the same F-actin rearrangement and Hirano body formation (Maselli et al., 2002). This indicates that *Dictyostelium* and mammalian cells use similar pathways to form Hirano bodies. The delay in *Dictyostelium* development in the presence of CT also suggests that CT causes cellular defects. Further characterization of the *Dictyostelium* model of Hirano bodies revealed that Hirano bodies can be cleared from the cell through both the autophagy and ubiquitin-proteasome degradation pathways (Kim et al., 2009). Additionally, mass spectrometry performed on partially purified Hirano bodies from *Dictyostelium* identified numerous proteins involved, including proteins involved with the cytoskeleton (Dong et al., 2016). Of these, four proteins were further investigated in the context of model Hirano bodies: profilin, actin-related protein (Arp) 2/3, vasodilator-stimulated phosphoprotein (VASP), and Wiskott-Aldrich Syndrome protein and scar homolog (WASH). Hirano bodies were unable to form under Arp2/3 inhibition and in cells lacking VASP or HSPC300, a protein involved in the WAVE (Wiskott-Aldrich Syndrome protein family verprolin-homologous protein) complex and activator of Arp2/3. This suggests that Hirano bodies require *de novo* actin polymerization to form in *Dictyostelium* (Dong et al., 2016). Because *Dictyostelium* can be used as an inducible model of Hirano bodies by expression of CT, it can be useful for further characterization of Hirano bodies as well as observation of long-term effects Hirano bodies may have on cellular functions. These studies can also be validated in mammalian systems expressing CT to help us better understand how Hirano bodies form and the effects they have on cellular processes.

### Mitochondrial Dysfunction

Mitochondria perform critical functions including metabolism and ATP production, reduction-oxidation control, and free-radical scavenging (Reddy, 2007, 2009). Because the brain has a high energy demand, mitochondrial function is critical to neuronal health (Han et al., 2021). Mitochondrial dysfunction has been observed in many neurodegenerative diseases including AD, PD, and ALS; however, it is unclear if mitochondrial dysfunction is a cause or byproduct of neurodegenerative diseases (Reddy and Beal, 2005; Manczak et al., 2006; Viscomi et al., 2016; Onyango et al., 2017; Pearce et al., 2019). In many familial neurodegenerative disease cases, mutant proteins such as Aβ in AD, parkin, DJ-1, and α-synuclein in PD, huntingtin in HD, and superoxide dismutase 1 (SOD1) in ALS can localize to the mitochondria. This has been suggested to cause a decrease in ATP production as well as an increase in free radical production, leading to degeneration (Beal, 2005; Reddy and Beal, 2008; Reddy, 2009). In PD, there are numerous mitochondrial genes mutated in familial cases that correspond to dysfunction including DJ-1, LRRK2, PRKN, PINK1, and HTRA2 (Kalinderi et al., 2016; Narendra, 2016; Pearce et al., 2019; Han et al., 2021). However, it is still largely unknown if and how mutations in neurodegenerative proteins exert toxicity on mitochondria. Aging also contributes to changes observed in mitochondrial function. Over time, defects in mitochondrial DNA accumulate and results in increased production of reactive oxygen species, which are ultimately involved in late-onset diseases and cell death (Swerdlow and Khan, 2004; Beal, 2005; Lin and Beal, 2006; Reddy, 2008; Reddy and Beal, 2008). However, why some individuals are more susceptible to late-onset neurodegenerative diseases than others is still unknown.

*Dictyostelium* mitochondrial pathways are similar to those of most eukaryotes and have many homologous proteins to mammalian mitochondrial proteins. Importantly, the oxidative phosphorylation pathway is the same between *Dictyostelium* and mammalian systems (Pearce et al., 2019). This makes *Dictyostelium* an attractive model for studying mitochondrial toxicity of proteins implicated in mitochondrial dysfunction in neurodegenerative diseases. *Dictyostelium* is a well-studied model of mitochondrial dysfunction. In *Dictyostelium*, mitochondrial dysfunction is defined by specific phenotypes including impaired phototaxis and thermotaxis, growth defects in axenic medium (pinocytosis) and on bacterial lawns (phagocytosis), chronic activation of AMP kinase, shorter and thicker stalks due to increased cell differentiation into pre-stalk cells, and altered ability to transition from growth to development (Bokko et al., 2007; Francione et al., 2011). *Dictyostelium* can therefore be easily used to investigate neurodegenerative disease proteins in the context of mitochondrial dysfunction. *Dictyostelium* encodes for many homologs of neurodegenerative disease proteins implicated in mitochondrial dysfunction, meaning they can be easily mutated or deleted to study their effects on mitochondria, such as with DJ-1 (Chen et al., 2017, 2021). However, there are some neurodegenerative disease proteins that do not have homologs in *Dictyostelium*, in which case these proteins can be introduced into *Dictyostelium* and their effects observed, such as with α-synuclein (Fernando et al., 2020). Overall, *Dictyostelium* provides a simple and useful model for studying the effects of neurodegenerative disease proteins on mitochondrial function.

## Homologous Neurodegenerative Disease Genes and Proteins Not Yet Studied in *Dictyostelium*

In addition to the studies discussed above, there are over 50 homologous neurodegenerative disease genes expressed in *Dictyostelium* (Table 1). Investigation of these genes in *Dictyostelium* will likely uncover novel aspects of their function. While *Dictyostelium* does not contain a complex nervous system that will uncover all aspects of their functions, it does have a more simplified genome that may result in decreased layers of genetic redundancy, unveiling novel aspects of gene function that may be missed in more complex organisms. Additionally, due to its larger genome, *Dictyostelium* expresses approximately 30 more neurodegenerative disease proteins than *S. cerevisiae*, allowing for investigation of the role of these proteins in a single-celled organism (Table 2).

Of the genes previously mentioned, *PSEN1, PSEN2*, *DJ-1, HTT, CLN3, CLN5*, and *TPP1 (CLN2)* are present in *Dictyostelium* but not *S. cerevisiae.* There are also four other AD-related genes, one other PD-related gene, and three other NCL-related genes expressed in *Dictyostelium* that have yet to be investigated in *Dictyostelium*. In addition, many other neurodegenerative diseases have the potential to be studied using *Dictyostelium* as a model organism. For example, *Dictyostelium* expresses genes implicated in ALS and some spinocerebellar ataxias. *Dictyostelium* also has homologous genes involved in other rare neurodegenerative diseases including Niemann-Pick, Refsum, and Tay-Sachs diseases, among others (Table 2). Notably, these genes are not expressed in *S. cerevisiae*, making *Dictyostelium* an advantageous single-cell model for studying the functions of these proteins. *Dictyostelium* can be utilized to learn about both normal and mutant functions of these proteins and consequently interrogate the pathways involved in the pathogenesis of their respective diseases.

## The Polyglutamine Diseases and *Dictyostelium’s* Polyglutamine/Asparagine-Rich Proteome

One unique aspect of *Dictyostelium’s* genome is that it has a remarkably large number of single sequence repeats (SSRs). Interestingly, many of these SSRs are present in protein-coding regions of genes, resulting in *Dictyostelium* encoding nearly 10,000 homopolymeric amino acid tracts. Surprisingly, there are homopolymeric tracts for every amino acid except tryptophan, with asparagine (N) and glutamine (Q) being the most abundant repeats (Eichinger et al., 2005). This is surprising because polyQ tracts cause a class of nine neurodegenerative diseases and *Dictyostelium* naturally encodes long polyQ repeats that are well beyond the disease threshold of ∼40 glutamines (Eichinger et al., 2005; Santarriaga et al., 2015). In addition to long polyQ repeats, there are numerous *Dictyostelium* proteins that have Q/N-rich sequences and are described as prion-like (Malinovska et al., 2015). Prions are proteins that can misfold and subsequently become transmissible. Other cells can then be infected by the transmission of misfolded prions, and this can impress the misfolded conformation on the normal proteins, causing prion diseases (Hope et al., 1986; Come et al., 1993; Cohen et al., 1994; Gajdusek, 1996; Harris, 1999; Bolton and Bendheim, 2007). Yeast has been commonly used as a model to study prion diseases as it expresses several prion proteins that are transmissible after misfolding (Liebman and Chernoff, 2012). The sequences of yeast prion proteins are Q/N-rich, making them more prone to misfolding and aggregation (Balch et al., 2008; Alberti et al., 2009; Halfmann et al., 2010, 2012). Prion-like sequences are also found in some aggregation-prone human neurodegenerative disease proteins (Gitler and Shorter, 2011; King et al., 2012; Kim et al., 2013). Surprisingly, Q/N-rich sequences naturally encoded in *Dictyostelium* do not aggregate, and it is unknown whether these *Dictyostelium* proteins share prion biology (Malinovska et al., 2015).

To date, only a few studies have investigated the roles that proteins with long amino acid tracts serve in *Dictyostelium* biology. Gene ontology annotation of the polyN and polyQ proteins revealed these repeats are enriched in protein kinases, lipid kinases, transcription factors, RNA helicases, and mRNA binding proteins associated with the spliceosome (Eichinger et al., 2005). Further bioinformatic analysis and gene ontology on proteins in *Dictyostelium* with Q/N-rich, prion-like sequences revealed that these sequences are linked to both proteinase K-like domains and RNA-binding domains (Malinovska et al., 2015). Prion-like domains were also found to be enriched in proteins associated with DNA/RNA interactions, protein modification, and signaling processes. It was concluded that these Q/N-rich sequences are not randomly occurring, but rather are conserved within the same protein families across *Dictyostelid* species (Malinovska et al., 2015). This suggests that the polyQ/N proteins may serve important biological functions related to DNA replication, transcription, and protein modification. However, research regarding the roles of homopolymeric repeats in *Dictyostelium* has not been readily pursued and the functions these polyQ/N proteins play are still largely unknown. It is intriguing that these proteins could be functional in *Dictyostelium*, and therefore future studies should be dedicated to learning more about the purpose of these proteins in *Dictyostelium* biology.

## *Dictyostelium* is Naturally Resistant to Polyglutamine Aggregation

**FIGURE 5:**
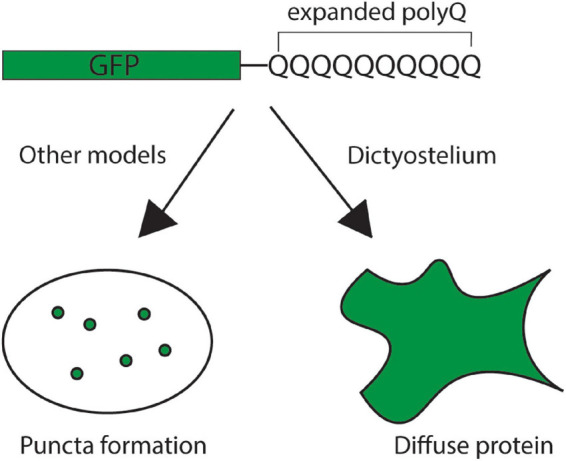
*Dictyostelium* is resistant to polyglutamine aggregation. Expression of an exogenous, pathogenic GFP-tagged mutant huntingtin exon 1 protein with an expanded polyglutamine tract yields the formation of polyglutamine puncta in the cells of most model organisms, indicating protein aggregation (*left*). In *Dictyostelium*, however, the mutant huntingtin protein remains diffuse throughout the cell, indicating that the protein remains soluble (*right*).

In addition to glutamine-rich regions forming prions, proteins with long polyQ tracts (>35Q) also cause a class of nine neurodegenerative diseases called the polyQ diseases. In these diseases, polyQ tracts within the coding region of specific genes become expanded resulting in aggregation-prone proteins that are neurotoxic. This led to the question: Is *Dictyostelium* naturally resistant to polyQ aggregation? Interestingly, unlike other model organisms, *Dictyostelium* is resistant to aggregation of mutant huntingtin exon 1 with 103 glutamines (Figure 5; Malinovska et al., 2015; Santarriaga et al., 2015). This is surprising because this fragment is highly aggregation-prone in other model organisms (DiFiglia et al., 1997; Li and Li, 1998; Krobitsch and Lindquist, 2000; Meriin et al., 2002; Santarriaga et al., 2015). To begin understanding how *Dictyostelium* resists protein aggregation a restriction enzyme mediated integration (REMI) screen was utilized to identify genes that are necessary for suppressing polyQ aggregation in *Dictyostelium*. This screen identified one gene that encodes for serine-rich chaperone protein 1 (SRCP1). Interestingly, SRCP1 is both necessary for suppressing polyglutamine aggregation in *Dictyostelium* and sufficient to suppress polyQ aggregation in other organisms (Santarriaga et al., 2018). One caveat of this screen was that it did not approach genome-wide coverage and additional suppressors of polyQ aggregation in *Dictyostelium* likely exist. The development of novel screening pipelines will enable genome-wide coverage in future screens, fully elucidating the genes that are essential for suppressing polyQ aggregation in *Dictyostelium* (Williams et al., 2021).

*Dictyostelium’s* resistance to polyQ aggregation raises many questions about how protein quality control pathways are regulated in *Dictyostelium*. In the future, studies investigating *Dictyostelium’s* response to various stressors are warranted and may identify novel aspects that regulate protein quality control. Initial work has identified heat shock protein 101 (Hsp101) as a key suppressor of polyQ aggregation during heat stress (Malinovska et al., 2015). However, the proteins and pathways that are necessary for suppressing protein aggregation during various states of stress in *Dictyostelium* are unknown. Identification of novel aspects of *Dictyostelium* protein quality control may lead to new insights into how *Dictyostelium* maintains proteostasis of its repeat-rich proteome during cellular stress.

Finally, understanding how *Dictyostelium* resists polyQ aggregation may lead to the development of novel therapeutics to treat neurodegenerative diseases. Toward this end, it was found that in addition to polyQ, SRCP1 can also suppress aggregation of superoxide dismutase 1 (SOD1) in human cells. Furthermore, SRCP1 was packaged in adeno-associated virus 9 (AAV9) and injected into the cortex of a mouse model of ALS. Expression of SOD1 in this mouse model resulted in decreased SOD1 aggregation in the cortex, however, it did not result in an increase in lifespan (Luecke et al., 2021). In the future, it will be important to deliver SRCP1 directly to affected neuronal populations to determine if SRCP1 provides neuroprotective properties in models of neurodegeneration.

## Conclusion

Neurodegenerative diseases are incurable diseases with few treatment options. In many neurodegenerative diseases, the proteins that are mutated or accumulate have unknown functions. Simple model organisms including *Dictyostelium discoideum* provide a platform to investigate the normal function of these proteins and to perform genetic screens to identify genes that modulate their functions. Because *Dictyostelium* is a simple model organism with a single cellular stage and encodes for numerous proteins implicated in neurodegenerative diseases, it is useful for understanding the normal function of these proteins and for identifying pathways that are disrupted in disease states. For example, knockout screens can easily be employed to determine the functions of the many homologous neurodegenerative disease proteins expressed in *Dictyostelium.* Additionally, the introduction of normal and mutant neurodegenerative disease genes not encoded for in *Dictyostelium’s* genome could also serve to identify protein functions and toxicity as well as cellular pathways affected. *Dictyostelium* may also be used to identify novel protein interactors with neurodegenerative disease proteins. Another important observation that must be further studied is *Dictyostelium’s* resistance to protein aggregation. Elucidating *Dictyostelium’s* proteostatic pathways could allow us to observe how *Dictyostelium* evolutionarily overcame the obstacle of protein aggregation. In the future, *Dictyostelium* can continue to serve as a simple, yet powerful, model to investigate neurodegenerative diseases and expand our knowledge to treat these diseases.

## Author Contributions

HH wrote the initial draft. HH and KS conceptualized and edited the review, contributed to the article, and approved the submitted version.

## Conflict of Interest

The authors declare that the research was conducted in the absence of any commercial or financial relationships that could be construed as a potential conflict of interest.

## Publisher’s Note

All claims expressed in this article are solely those of the authors and do not necessarily represent those of their affiliated organizations, or those of the publisher, the editors and the reviewers. Any product that may be evaluated in this article, or claim that may be made by its manufacturer, is not guaranteed or endorsed by the publisher.
